# Herpes Simplex Virus 1 Induces Microglia Gasdermin D-Dependent Pyroptosis Through Activating the NLR Family Pyrin Domain Containing 3 Inflammasome

**DOI:** 10.3389/fmicb.2022.838808

**Published:** 2022-03-21

**Authors:** Xiao Hu, Qiongzhen Zeng, Ji Xiao, Shurong Qin, Yuan Wang, Tianhao Shan, Di Hu, Yexuan Zhu, Kaisheng Liu, Kai Zheng, Yifei Wang, Zhe Ren

**Affiliations:** ^1^Guangzhou Jinan Biomedical Research and Development Center, College of Life Science and Technology, Institute of Biomedicine, Jinan University, Guangzhou, China; ^2^The Key Laboratory of Virology of Guangdong, Jinan University, Guangzhou, China; ^3^Department of Cell Biology, College of Life Science and Technology, Jinan University, Guangzhou, China; ^4^College of Pharmacy, Jinan University, Guangzhou, China; ^5^Guangdong Province Key Laboratory of Bioengineering Medicine, Jinan University, Guangzhou, China; ^6^National Engineering Research Center of Genetic Medicine, Jinan University, Guangzhou, China; ^7^Shenzhen People’s Hospital (The Second Clinical Medical College, Jinan University, The First Affiliated Hospital, Southern University of Science and Technology), Shenzhen, China; ^8^School of Pharmaceutical Sciences, Health Science Center, Shenzhen University, Shenzhen, China

**Keywords:** pyroptosis, inflammation, NLRP3 inflammasome, Gasdermin D, Herpes simplex virus type 1, herpes simplex virus encephalitis

## Abstract

Herpes simplex virus type 1 (HSV-1) is a highly prevalent virus in humans and causes severe forms of inflammation, such as herpes simplex encephalitis (HSE). Pyroptosis is a new inflammatory cell death triggered by inflammasome and cysteine-requiring aspartate protease-1 (caspase-1) activation. Nonetheless, HSV-1 induces encephalitis, and cell death mechanisms are not understood. In this study, we confirmed for the first time that the DNA virus HSV-1 triggers Gasdermin D-dependent pyroptosis by activating NLR family pyrin domain containing 3 (NLRP3) inflammasomes in mouse microglia, leading to mature IL-1β production and active caspase-1 (p10) release. Inhibition of microglial NLRP3 inflammasome activation suppressed HSV-1-induced Gasdermin D-dependent pyroptosis. In addition, NLRP3 and IL-1β expression levels were significantly increased in the mouse model of herpes simplex encephalitis compared with normal mice without viral infection. Collectively, our data revealed that the activation of inflammasomes and GSDMD-dependent pyroptosis is the mechanism of HSV-1 inducing inflammation and provides treatment targets for viral inflammation.

## Introduction

Herpes simplex encephalitis (HSE), the fatal encephalitis in humans, is caused by Herpes simplex virus type 1 (HSV-1) (Whitley, 2006). Herpes simplex virus type 1 causes a series of powerful immunological inflammatory reactions, which leads to pathological damage to brain tissue (Esiri et al., 1995; Kennedy and Chaudhuri, 2002; Sergerie et al., 2007). As resident cells of the central nervous system, microglia can respond quickly to external pathogens and tissue damage (Plüddemann et al., 2011). After HSV-1 infection, microglia release various cytokines and have the dual effects of anti-virus and immune destruction (Conrady et al., 2010; Chen et al., 2019; Piret and Boivin, 2020). Recently, some studies have indicated that high levels of multiple inflammatory cytokines and cell death can be found *in vivo* and the brains of herpes simplex encephalitis (Guo et al., 2010; Laukoter et al., 2017). Most research on HSV-1 and cell death has focused on apoptosis (Sanfilippo et al., 2004; Aurelian, 2005; Sanfilippo and Blaho, 2006; He Q. et al., 2020). However, as a type of programmed cell death, apoptosis is notable for being non-inflammatory. It is worth noting that pyroptosis has a strong link to inflammatory diseases. The evidence supports that HSV-1 infection might cause inflammatory cell death.

Pyroptosis, also known as cell inflammatory cell death, is defined by the persistent enlargement of cells until the cell membrane ruptures, allowing the cell’s contents to release and triggering severe inflammation (Shi et al., 2017). Pyroptosis is intimately linked to the activation of inflammasomes, the activation of inflammasomes, the cleavage of Gasdermin D (GSDMD), and the release of many proinflammatory factors are all biochemical features of pyroptosis (Man et al., 2017). Nucleotide-binding oligomerization domain (NOD)-like receptors in the cytoplasm can recruit pro-caspase-1 directly or through the adaptor protein apoptosis-associated speck-like protein containing a caspasedomain (ASC) to active inflammasomes when cells are infected by external pathogens (such as viruses or bacteria) (He et al., 2016). The inflammasome forms active cysteine-requiring aspartate protease-1 (caspase-1), which causes mature IL-1β and IL-18. Simultaneously, an active amino-terminal (GSDMD-N) is produced, promoting cell membrane perforation and the release of mature IL-1β and IL-18 into the extracellular environment, exacerbating inflammation (Swanson et al., 2019). It is still uncertain whether HSV-1 causes macrophage pyroptosis and exacerbates inflammation. In this study, our results indicate that HSV-1 induces GSDMD-dependent microglia pyroptosis via the NLR family pyrin domain containing 3 (NLRP3) inflammasome activation. These experimental findings help improve the understanding of the relationship between HSV-1, pyroptosis, and inflammasome in the pathogenesis of herpes simplex encephalitis.

## Materials and Methods

### Cell Culture and Viruses

Mouse microglial cells (BV2), human microglia cells (HMC3), and mouse macrophage cells (RAW264.7) were purchased from the Cell Bank of the Chinese Academy of Sciences (Shanghai, China) and cultured in DMEM with 10% FBS (Thermo, Gibco, 10270106, United States). African green monkey kidney cells (Vero) were purchased from American Type Culture Collection (ATCC) and cultured in DMEM with 10% FBS (Hangzhou Sijiqing Biology Engineering Materials Co., Ltd., Hangzhou, China). All the cells were cultured at 37°C in a humid atmosphere with 5% CO_2_. HSV-1 F strain (ATCC, United States) is a gift from the University of Hong Kong and is stored at –80°C.

### *In vitro* Infection

BV2, HMC3, and RAW264. 7 were seeded in a 60 mm Petrie dish at 8 × 10^6^ cells per dish. At 12 h after seeding, after 1 h of drug pretreatment, a mixed medium of virus and drug was added. After the time point required for infection, samples were collected.

### Quantitative Real-Time PCR

According to the manufacturer’s instructions, total RNA was extracted from cells and mouse brain tissue (Trizol, DP424) and was reverse transcribed using the Reverse transcriptase (Accurate Biology, AG11706). The qRT-PCR was performed using a Bio-Rad CFX96 real-time PCR system with SYBR Green Realtime PCR Master Mix (Accurate Biology, AG11702). Data were processed and analyzed using Bio-Rad CFX Manager software. Mouse β-actin was used as an internal control for data normalization. The sequences of the primers used are provided in Supplementary Table 1.

### Western Blot Analysis

The same techniques described elsewhere were used to extract total protein from cells and mice. The BCA Protein Assay kit was used to determine the protein concentrations (P0009, Beyotime, China). The cell lysates were mixed with 5 × SDS-PAGE buffer (P0015, Beyotime, Shanghai, China) and boiled for 10 min. Subsequently, the proteins were subjected to 10% SDS-PAGE and transferred onto polyvinylidene fluoride membranes (ISEQ00010, Millipore, United States). After sealing with 5% skim milk for more than 1 h, the membranes were incubated with the primary antibodies against NLRP3 (CST, 15101S), Caspase-1 (Abcam, ab179515), IL-1β (CST, 12242S), ASC (CST, 67824S), β-actin (CST, 4970S), gB (Santa, sc-56987), GSDMD (Proteintech, 20770-1-AP), GSDMD (Abcam, ab209845), and C-terminal GSDMD (Abcam, ab255603) at 4°C overnight. After washing with TBST three times, the membranes were incubated with the fluorescence-conjugated IgG secondary antibody (1:6000) for 1 h.

### Enzyme-Linked Immunosorbent Assay

The protein levels were detected by an ELISA kit according to the manufacturer’s protocols (Purchased from 4A biotech, mouse IL-1β, CME0015).

### Cell Death Assay

BV2 cells were inoculated in a six-well plate for 12 h and then Mock and HSV-1 infection. After 24 h of infection, pyroptotic and apoptotic cell death were evaluated with PI staining (2 μl/mL)/Hoechst 33342 (5 μl/mL) and Annexin V-APC (6 μl/mL)/Hoechst33342 (5 μl/mL) (Zha et al., 2016). PI staining (#638) and Hoechst 33342 (#639) were purchased from immunochemistry. Annexin V-APC was purchased from KGI Biosciences. Dead cells (Annexin V-APC and PI permeable) were determined under a Nikon light microscope (Eclipse Ti-E) and analyzed with NIS-Elements Viewer 4.50 and ImageJ program.

### Lactate Dehydrogenase Cytotoxicity Assay Kit

Lactate dehydrogenase (LDH) Cytotoxicity Assay Kit was purchased from Beyotime (C0017). According to the manufacturer’s recommended protocol, 0.5% heat-inactivated 10% FBS + DMEM and the LDH were released into the cell culture supernatant after different treatments were measured. In short, inoculate BV2 in a 12-well plate for 12 h. The next day, the cells are infected with mock or HSV-1, and the supernatant is collected. Cytotoxicity or mortality (%) = (absorbance of processed sample-absorbance of sample control well)/(absorbance of cell maximum enzyme activity-absorbance of sample control well) × 100%.

### Animals

The BABL/C (4–5 weeks, male) mice were brought from Guangdong Medical Experimental Animal Center (China). Mice were infected with the 2 × 10^6^ plaque formation units (PFUs) of HSV-1 by nasal drops.

### Statistical Analysis

All experiments were performed three times independently, with one representative experiment shown. GraphPad Prism 8 statistical software was used to analyze the data. The experimental data were assessed by Student’s *t*-test, one-way ANOVA. Data were expressed as mean ± SD. *P* < 0. 05 was considered statistically significant. *P*-values of < 0.05, < 0.01, and < 0.005 were marked as *, ^**^, and ^***^ separately.

## Results

### Herpes Simplex Virus Type 1 Promotes Cell Death in Cultured BV2

**FIGURE 1:**
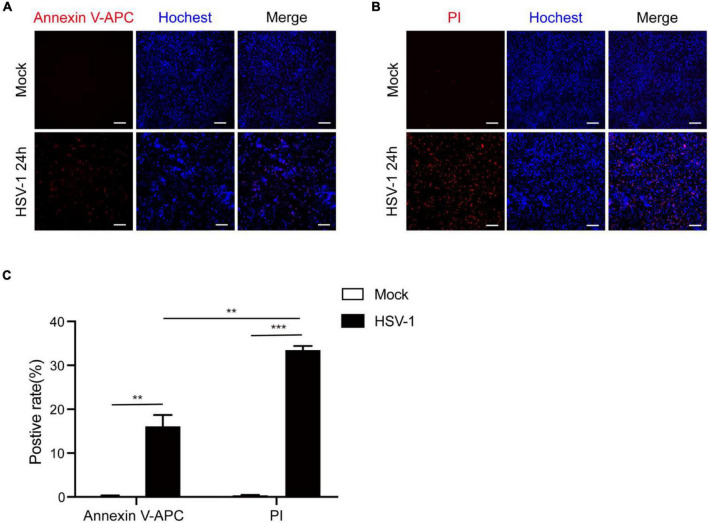
HSV-1 induces cell death in cultured BV2. **(A)** Representative micrographs of Annexin V-APC and Hoechst 33342 staining of BV2 with Mock or HSV-1 (MOI = 5) infection. Red: Annexin V-APC; blue: Hoechst 33342 dye (Scale bar, 100 μm, 10×). **(B)** Representative micrographs of propidium iodide (PI) and Hoechst 33342 staining BV2 cells with Mock or HSV-1 (MOI = 5) infection. Red: PI dye; blue: Hoechst 33342 dye (Scale bar, 100 μm, 10 ×). **(C)** Quantification of the number of PI + cells and Annexin V-APC + cells relative to mock and HSV-1 infection are shown in the histogram. All data are presented as mean ± SD, Student’s *t*-test, **P < 0.01, ***P < 0.001.

According to previous research, most investigations into HSV-1 and cell death have focused on apoptosis. After infection, HSV-1 can induce caspase-3-mediated apoptotic cell death, and HSV-1, but not HSV-2, causes apoptosis in adult CNS neurons (Gautier et al., 2003; Aurelian, 2005; Sanfilippo and Blaho, 2006). Based on our observations of inflammatory damage in the brains of herpes simplex encephalitis, apoptosis is a type of non-inflammatory cell death. Furthermore, the RNA virus has been shown to cause pyroptosis in brain cells and increase neuroinflammation (He X. et al., 2020; He Z. et al., 2020). We speculate that HSV-1 causes inflammation-related cell death to aggravate inflammation. Microglia play a critical function in regulating the immunological environment of the central nervous system. The Annexin V-APC/Hoechst33342 and PI/Hoechst33342 double staining methods were used to count the positive rates of apoptosis and pyroptosis in BV2 cells after HSV-1 infection (Figures 1A,B) to evaluate whether HSV-1 induces cell death. As expected, HSV-1 induced pyroptosis and apoptosis in microglia. As shown in Figure 1C, the BV2 cell apoptosis rate and pyroptosis rate are 16. 1 and 33. 5%, respectively. Our findings indicate that HSV-1 induced pyroptotic cell death in BV2.

### Herpes Simplex Virus Type 1 Infection Induces Pyroptosis and Triggers Interleukin 1β Maturation

**FIGURE 2:**
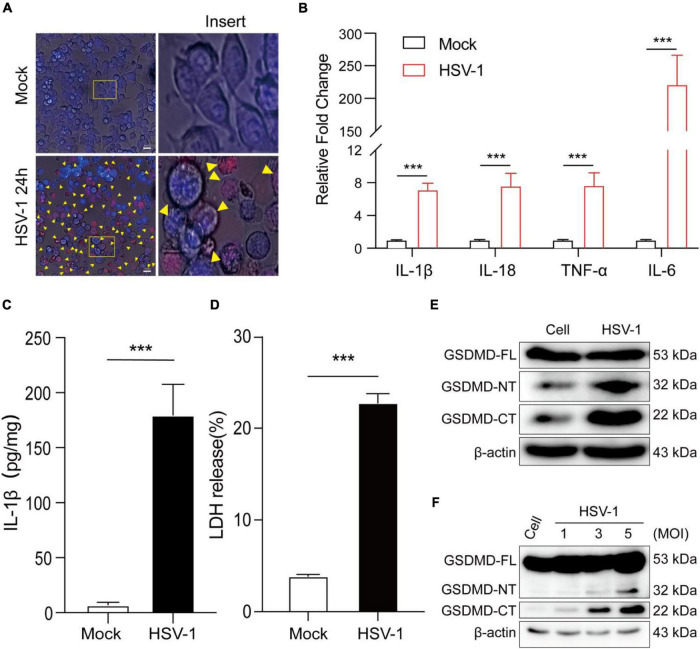
HSV-1 infection induces pyroptosis and triggers interleukin 1β (IL-1β) maturation. **(A)** Representative micrographs of BV2 cells infected with HSV-1 (MOI = 5, 24 hpi) or mock infection. Yellow corner mark: pyroptotic cells (Scale bar, 100 μm, 40×). **(B)** Relative qRT-PCR analysis of IL-1β, IL-18, TNF-α, and IL-6 mRNA levels in BV2 cells infected with HSV-1 and Mock infection (MOI = 5, 24 hpi). **(C)** IL-1β was measured with ELISA in BV2 with HSV-1 and mock infection (MOI = 5, 24 hpi). **(D)** LDH release was measured in supernatant taken from cultured mock- and HSV-1-infected BV2. **(E,F)** Examination of the proteolytic cleavage of GSDMD in BV2 infected by HSV-1 for the indicated MOI (24 hpi), Western blot analysis of indicated proteins in the cell lysates. GSDMD-FL, full-length GSDMD; GSDMD-NT, the N-terminal cleavage product of GSDMD; GSDMD-CT, the C-terminal cleavage product of GSDMD. β-actin was used as a loading control. All data are presented as mean ± SD, Student’s *t*-test, ***P < 0. 001.

To further understand HSV-1 induced pyroptosis, we next observe the cell morphology after HSV-1 infection. Morphological examination found that HSV-1 infected BV2 cells have typical cell swelling and membrane rupture, but it was not found in the simulated mock-infection (Figure 2A). Next, the mRNA expression levels of inflammatory cytokines, including IL1β, IL-18, TNF-α, and IL-6, were examined, which showed that all the genes were significantly increased by HSV-1 infection (Figure 2B). HSV-1 could significantly induce secretion of the inflammatory cytokine IL-1β in BV2 (Figure 2C). Lactate dehydrogenase (LDH) activity was evaluated in the cell culture supernatant to evaluate the integrity of the cell membrane during pyroptosis (Liu et al., 2019). HSV-1 infection caused a significant increase in LDH release in BV2 cells and HMC3 cells (Figure 2D and Supplementary Figure 1A). GSDMD was identified as the executor of the pyroptosis (Shi et al., 2015; Kovacs and Miao, 2017). HSV-1 infection can induce proteolytic cleavage of GSDMD in BV2 cells and HMC3 cells (Figure 2E and Supplementary Figure 1B). The cleavage of GSDMD is dose-dependent with the HSV-1 MOI value (Figure 2F). Consistently, HSV-1 causes trigger GSDMD-dependent pyroptosis and stimulates the release of the inflammatory cytokine IL-1β *in vitro*.

### NLR Family Pyrin Domain Containing 3 Inflammasome Is Involved in Pyroptosis Caused by Herpes Simplex Virus Type 1

**FIGURE 3:**
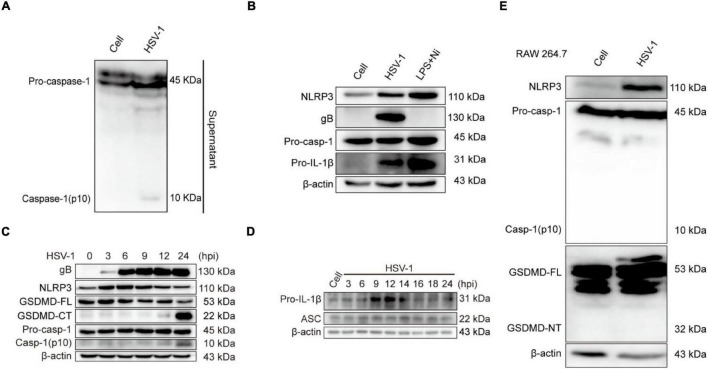
NLRP3 Inflammasome is involved in HSV-1-induced pyroptosis. **(A)** Western blot analysis of indicated proteins in the supernatants (Sup), BV2 infected with HSV-1 (MOI = 5, 24 hpi). **(B)** BV2 cells treated with 1 μg/mL lipopolysaccharides (LPS) for 4 h followed by 2 μg/mL nigericin for 2 h or HSV-1 infection (MOI = 5, 24 hpi), the level of NLRP3, pro-caspase-1, gB, and IL-1β was detected by Western blot. LPS + Nigericin in uninfected cells served as a positive control. **(C,D)** Immunoblot analysis of extracts of BV2 cells infected by HSV-1 (MOI = 5) for the indicated time points by the indicated antibodies. **(E)** The expression levels of NLRP3, caspase-1(p10), and cleavage of GSDMD expression in RAW264.7 were detected by Western blot. β-actin was used as a loading control.

The activation of inflammasomes in the innate immune response is an important mechanism for their anti-infective effects. Pattern recognition receptors (PRRs) recognize pathogens and cause the activation of inflammasomes (Bergsbaken et al., 2009; Man et al., 2017). The Nod-like family receptor member most studied with viruses is the NLRP3 inflammasome, activated NLRP3 recruits ASC and promotes the cleavage of pro-caspase-1 to create active caspase-1 (p10), resulting in the conversion of pro-IL-1β and pro-IL-18 to mature IL-1β and IL-18 (Lupfer and Kanneganti, 2013; Zheng, 2021). The current study only confirmed that the DNA virus HSV-1 upregulates the production of NLRP3 inflammasomes at the transcription and protein levels (Johnson et al., 2013; Karaba et al., 2020). We next investigated whether NLRP3 is involved in HSV-1-induced pyroptosis. The protein expression of caspase-1 (p10) in the supernatant was detected to verify if the inflammasomes are involved in HSV-1-induced pyroptosis (Figure 3A). After HSV-1 infection, NLRP3, viral protein gB, and pro-IL-1β were dramatically upregulated (Figure 3B and Supplementary Figures 2A,B). After HSV-1 infection at different times, caspase-1 (p10) release, GSDMD cleavage, and IL-1β expression are upregulated at 12 hpi (Figures 3C,D and Supplementary Figure 3A). LPS + Nigericin (LPS + Ni) was used as a positive control (He et al., 2015), LDH release rate increased significantly at 12 hpi (Supplementary Figure 3B). ASC is required to form inflammasomes and the recruitment of caspase-1 (Hoss et al., 2017). RAW 264. 7 lacks the ASC adaptor protein and cannot form the NLRP3 inflammasome complex (Pelegrin et al., 2008). After HSV-1 infection, Caspase-1 and GSDMD were not cleaved (Figure 3E). Overall, we suppose that NLRP3 inflammasome is involved in HSV-1 induced pyroptosis of BV2 cells but did not induce RAW 264. 7 pyroptosis.

### NLR Family Pyrin Domain Containing 3 Inhibitor MCC950 Prevents Herpes Simplex Virus Type 1-Induced Pyroptosis

**FIGURE 4:**
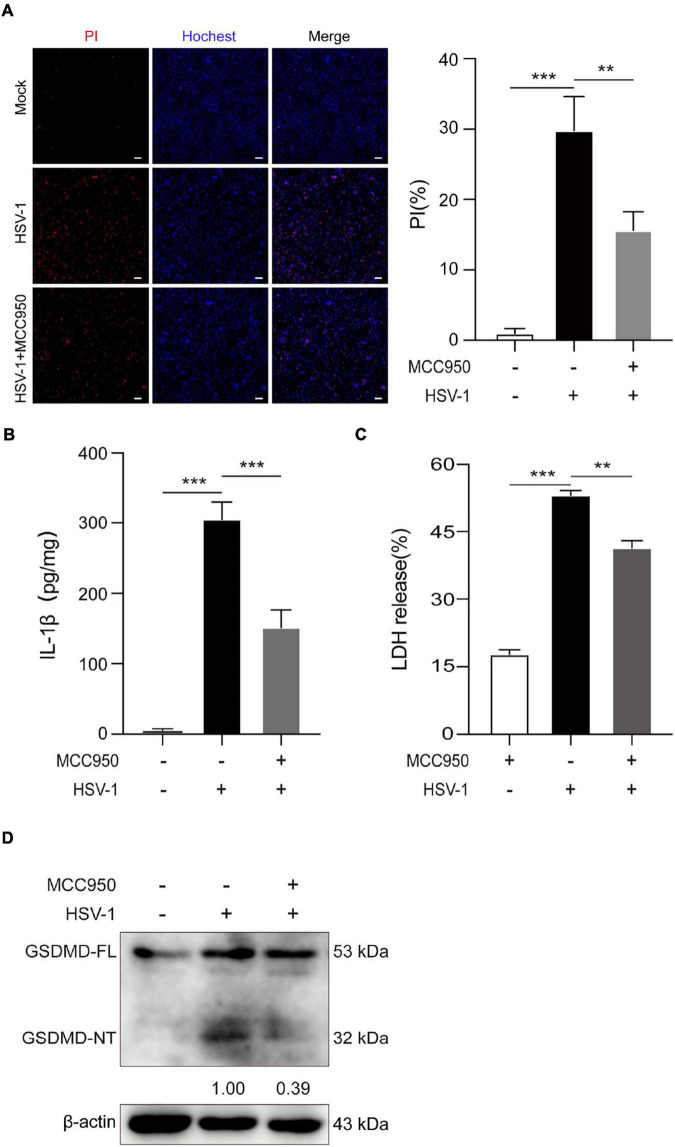
NLRP3-specific inhibitor MCC950 inhibits HSV-1 induced pyroptosis. **(A)** Cells were stained by Hoechst 33342 (blue; for all cells) and PI (red; for dead cells) for 10 min. All images were captured by fluorescence microscopy, and the merged images show PI and Hoechst 33342 fluorescence with bright-field images. One set of representative images of three independent experiments is shown (scale bars, 100 μm, 10×). PI-positive cells in five randomly chosen fields (one field per well) were quantified. The percentage of cell death is defined as the ratio of PI-positive relative to all (revealed by Hoechst 33342) cells. **(B)** ELISA of IL-1β in the supernatants of BV2 cells pretreated with MCC950 (5 μg/mL) for 1 h and subsequently infected with HSV-1 (MOI = 5) for 24 h. **(C)** BV2 cells pretreated with MCC950 (5 μg/mL) for 1 h and LDH release was measured in supernatant derived from MCC950, HSV-1 (MOI = 5) and HSV-1 + MCC950 (5 μg/mL). **(D)** BV2 cells were treated as **(C)** in the presence of MCC950, and the total DNA and protein of HSV-1 were extracted for analysis. Immunoblot analysis of extracts of BV2s by the indicated antibodies. All data are presented as mean ± SD, Student’s *t*-test, **P < 0.01, ***P < 0.001.

Further study the role of NLRP3 inflammasome in HSV-1 induced pyroptosis. MCC950 is a specific small-molecule inhibitor of NLRP3 inflammasome with a good ability to permeate the blood–brain barrier (Wu et al., 2020). MCC950 prevents ASC oligomerization generated by NLRP3 rather than preventing K + efflux, Ca 2 + flow, or NLRP3-ASC interaction (Coll et al., 2015). The PI/Hochest test revealed that MCC950 significantly decreased the HSV-1-induced PI-positive rate from 29.8 to 15.6% (Figure 4A). ELISA analyses revealed that HSV-1 infection induced the release of proinflammatory cytokines IL-1β. However, such an upregulation was inhibited by MCC950 treatment in BV2 cells (Figure 4B). Moreover, we found that MCC950 treatment suppressed the LDH release rate and the cleavage of GSDMD after HSV-1infection in BV2 cells and HMC3 cells (Figures 4C,D and Supplementary Figures 3A,B). In conclusion, these findings support the hypothesis that NLRP3 is involved in HSV-1-induced pyroptosis.

### Herpes Simplex Virus Type 1 Infection Triggers Severe Pathology *in vivo*

**FIGURE 5:**
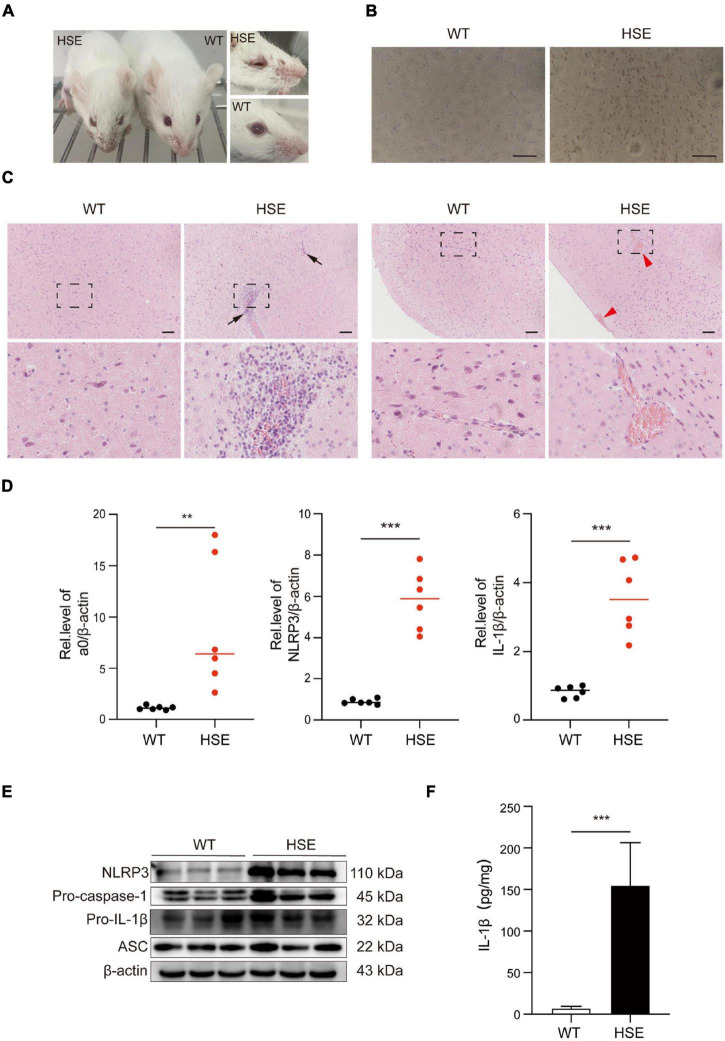
HSV-1 infection triggers severe pathology in the brain. **(A)** Photos of the front and eyes of mice in the normal and virus-infected groups. **(B)** Immunohistochemical micrographs of brain sections stained with HSV-1 (Scale bars, 100 μm). **(C)** Histopathological micrograph of H&E staining of the cerebral cortex (8 dpi). Black arrow: immune cell infiltration; red arrow: perivascular cuff (Scale bars, 100 μm). **(D)** The qRT-PCR method was used to detect the transcript levels of viral genes a0, NLRP3, and IL-1β in mouse brain specimens (8 dpi) (*n* = 6). **(E)** The protein of NLRP3, Pro-caspase-1, Pro-IL-1β, and ASC in mouse brain specimens was detected by Western blot analysis (8 dpi). **(F)** ELISA was used to detect IL-1β in mock-infected and HSV-1 infected mice sera. All data are presented as mean ± SD, Student’s *t*-test, **P < 0.01, ***P < 0.001.

We used mice to create an HSV-1 infection model *in vivo* to investigate further the pathological damage caused by HSV-1 infection in the mouse brain. The HSV-1 infected mice developed sores on their faces and eyes, and their fur was rough compared to the control. Immunohistochemistry (IHC) analysis for anti-HSV was used to assess viral infections in the brains. The above experimental results show that the mouse model of herpes simplex encephalitis has been effectively established (Figures 5A,B). Compared to wild-type mice, the HSV-1 infected mouse brains showed a large amount of neutrophil infiltration, necrosis, and destruction of normal cell structure, indicating severe focal inflammation in the brain (Figure 5C). The transcript levels of the α0, NLRP3, and the cytokine IL-1β were measured. The results revealed that the expression of NLRP3, α0, and IL-1β was upregulated in HSV-1 infected mice (Figure 5D). HSV-1 infection upregulated the expression of NLRP3 and IL-1β in animal tissue samples (Figure 5E). Furthermore, the expression levels of proinflammatory cytokines IL-1β were evaluated in serum, IL-1β secretion was stimulated by HSV-1 (Figure 5F). Our results showed that HSV-1 infection could produce severe inflammatory brain injury and activate the NLRP3 inflammasomes *in vivo*.

## Discussion

HSV-1 infection causes herpes simplex virus encephalitis (HSE), an inflammatory illness of the central nervous system. The mechanism behind this process, however, is still a mystery. Programmed cell death is an important host defense strategy to eliminate pathogen infection. Different cell death mechanisms are extremely complicated and might lead to excessive inflammatory responses. Virus-induced cell pyroptosis has rarely been studied compared with non-inflammatory cell apoptosis. Several studies on pyroptosis by viruses primarily focus on RNA viruses, such as the Zika virus, Influenza virus, Newcastle virus, HIV, and other RNA viruses, have been shown to activate NLRP3 inflammasomes cause inflammation (Wang et al., 2018; Gao et al., 2020; He X. et al., 2020; Xie et al., 2020). The release of proinflammatory cytokines IL-1β and IL-18 induced by inflammasome activation is a crucial component of the host’s innate immune response, and excessive secretion of proinflammatory cytokines exacerbates inflammation. We confirmed that HSV-1 induces Gasdermin D-dependent pyroptosis by activating NLRP3 inflammasomes *in vitro*, leading to mature IL-1β production and active caspase-1 (p10) release.

**FIGURE 6:**
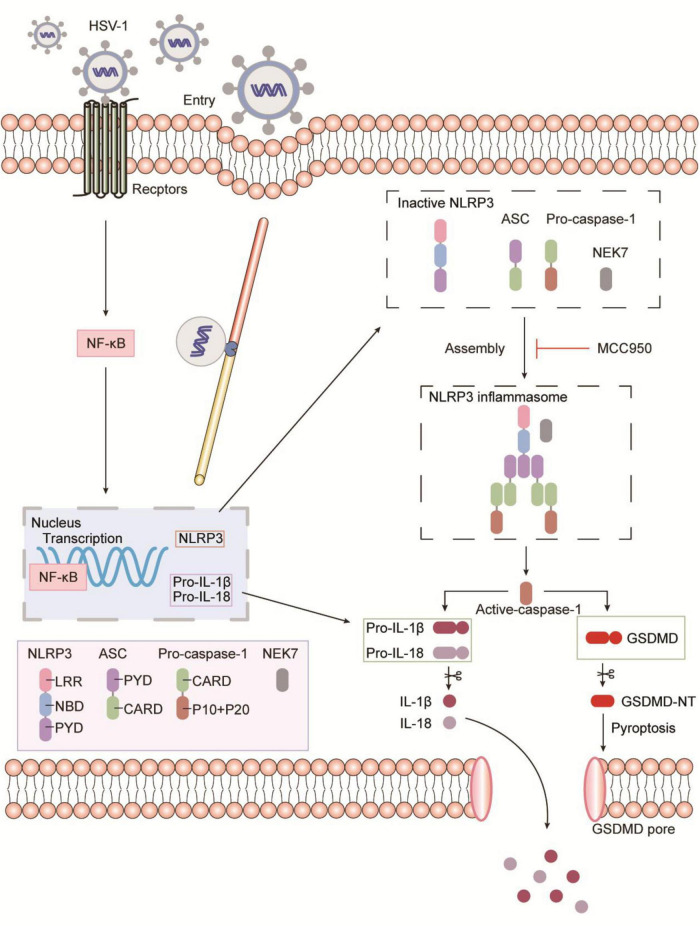
Schematic representation of HSV-1 induces microglia pyroptosis and inflammation by activating the NLRP3 inflammasome. During HSV-1infection, the pattern recognition receptor (PRR) activates the NF-κB signaling pathway, which causes the transcription and expression of critical proteins such as NLRP3, pro-IL-1β, and pro-IL-18. Caspase-1 cleavage, Gasdermin D-dependent pyroptosis, and IL-1β release occur after the NLRP3 inflammasome is formed. NLRP3 inflammasome selective inhibitor MCC950 inhibits HSV-1 induced Gasdermin D-dependent pyroptosis.

Moreover, NLRP3 inflammasome selective inhibitor MCC950 reduces virus-induced GSDMD-dependent pyroptosis and the release of IL-1β and LDH (Figure 4 and Supplementary Figure 4). It is worth noting that MCC950 has been shown to reduce the production of proinflammatory factors such as IL-1β, IL-18, TNF-α, and IL-6, and reduce the severity of inflammatory diseases (Coll et al., 2015; Perera et al., 2018; Xu et al., 2018). Based on MCC950 anti-inflammatory properties and good blood–brain barrier permeability in viral infections, inflammasome inhibitors can be used as a platform for the development and treatment of inflammatory diseases. As shown in Figure 6, the schematic diagram of HSV-1 induces microglia pyroptosis and inflammation by activating the NLRP3 inflammasome. Our experiments *in vivo* have shown that HSV-1 activates the NLRP3 inflammasome and promotes the release of IL-1β, consistent with the *in vitro* results (Figure 5). We suppose that pyroptosis would be potentially involved in HSV-1-induced herpes simplex encephalitis. Apoptosis is non-inflammatory cell death, whereas pyroptosis and necroptosis lead to the release of DAMPs such as IL-1β and HMGB1.

Interestingly, HSV-1 ICP6 initiates necroptosis in mouse cells, but it prevents necroptosis in human cells by inhibiting the interaction between receptor-interacting protein kinase 1 (RIP1) and RIP3 (Wang et al., 2014; Guo et al., 2015; Huang et al., 2015). The herpes simplex virus has evolved mechanisms to manipulate cell death (He and Han, 2020). Inflammatory cell death in herpes simplex encephalitis remains to be further investigated.

In summary, we showed that HSV-1 causes macrophage pyroptosis by inducing NLRP3 inflammasome activation, caspase-1, and Gasdermin D cleavage, and increased IL-1β production. These findings indicate that NLRP3 inflammasome activation induces pyroptosis. Death-related inflammatory chronic diseases provide a potential basis for treatment. To alleviate herpes simplex encephalitis, limiting inflammasome activation and macrophage pyroptosis and maintaining macrophage function are useful treatments. Our findings contribute to a better understanding of HSV-1-induced herpes simplex encephalitis and anti-pyroptosis-induced inflammatory disorders, as well as therapy strategies.

## Data Availability Statement

The original contributions presented in the study are included in the article/Supplementary Material, further inquiries can be directed to the corresponding authors.

## Author Contributions

XH and QZ formulated the idea of the article, performed the research, and analyzed the data. XH wrote the manuscript. JX, SQ, YuW, TS, YZ, DH, and KL revised the data and improved manuscript writing. KZ, ZR, and YiW were responsible for the planning and coordination of the research activity and the acquisition of the financial support for the project leading to this publication. All authors reviewed the manuscript and approved the final version of the manuscript.

## Conflict of Interest

The authors declare that the research was conducted in the absence of any commercial or financial relationships that could be construed as a potential conflict of interest.

## Publisher’s Note

All claims expressed in this article are solely those of the authors and do not necessarily represent those of their affiliated organizations, or those of the publisher, the editors and the reviewers. Any product that may be evaluated in this article, or claim that may be made by its manufacturer, is not guaranteed or endorsed by the publisher.

## Supplementary Material

Supplementary Figure 1 HSV-1 infection induces HMC3 pyroptosis. **(A)** LDH release was measured in supernatant taken from HMC3 infected by HSV-1

(MOI = 5) for 24 h. **(B)** HMC3 cells were infected with HSV-1 (MOI = 5) for 24 h, GSDMD-NT in the supernatants, and GSDMD-FL and β-actin in the cell lysates (Lys) were determined by western blot. All data are presented as mean ± SD, Student’s *t*-test, ****P* < 0.001.

Supplementary Figure 2 HSV-1 induces NLRP3 expression. **(A)** Relative qRT-PCR analysis of NLRP3 mRNA levels in BV2 cells infected with HSV-1(MOI = 5, 24 hpi). **(B)** HMC3 cells were infected with HSV-1 (MOI = 5) for 24 h, NLRP3 was detected by Western blot. All data are presented as mean ± SD, Student’s *t*-test, ****P* < 0.001.

Supplementary Figure 3 HSV-1 began to induce microglia pyroptosis at 12 hpi. **(A)** Immunoblot analysis of supernatants (Sup) of BV2 infected by HSV-1 (MOI = 5) for the indicated time points by the caspase-1 (p10). **(B)** LDH release was measured in the supernatant from simulated Mock and HSV-1 infected BV2 (12 hpi). All data are presented as mean ± SD, Student’s *t*-test, **P* < 0.05, ****P* < 0.001.

Supplementary Figure 4 MCC950 inhibits HSV-1 induced pyroptosis in HMC3 cells. **(A)** HMC3 cells pretreated with MCC950 (5 μg/mL) for 1 h and LDH release was measured in supernatant derived from MCC950, HSV-1 (MOI = 5) and HSV-1 + MCC950 (5 μg/mL). **(B)** HMC3 cells were treated as **(A)** in the presence of MCC950, and the total DNA and protein of HSV-1 were extracted for analysis. Immunoblot analysis of extracts of BV2s by the indicated antibodies. All data are presented as mean ± SD, Student’s *t*-test, ***P* < 0.01, ****P* < 0.001.
