# Supplementary figures and images for: Herpes Simplex Virus 1 Induces Microglia Gasdermin D-Dependent Pyroptosis Through Activating the NLR Family Pyrin Domain Containing 3 Inflammasome
Source: Front Microbiol. 2022 Mar 21;13:838808. doi: 10.3389/fmicb.2022.838808 (PMC8978634; doi:10.3389/fmicb.2022.838808)

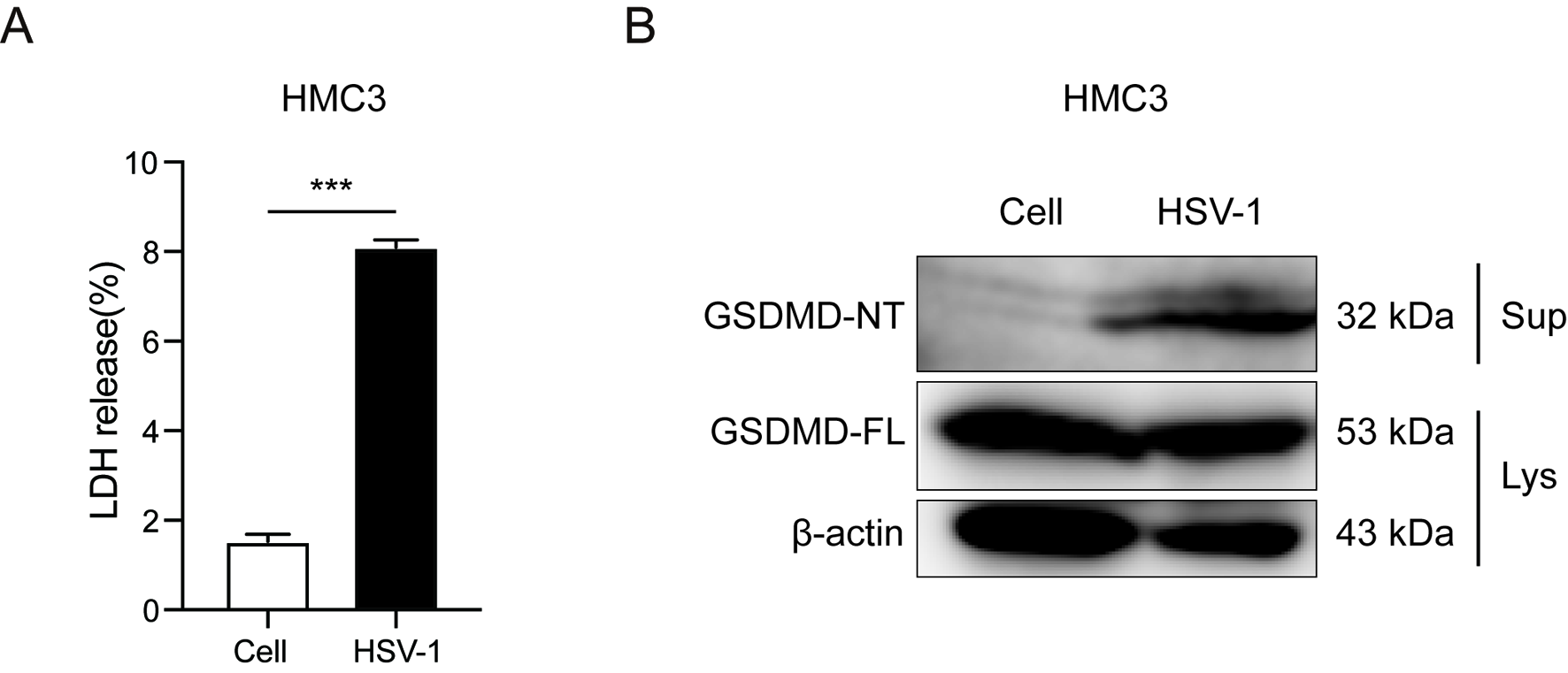

Supplement: Supplementary Figure 1 — HSV-1 infection induces HMC3 pyroptosis. (A) LDH release was measured in supernatant taken from HMC3 infected by HSV-1 [file Image_1.TIF]

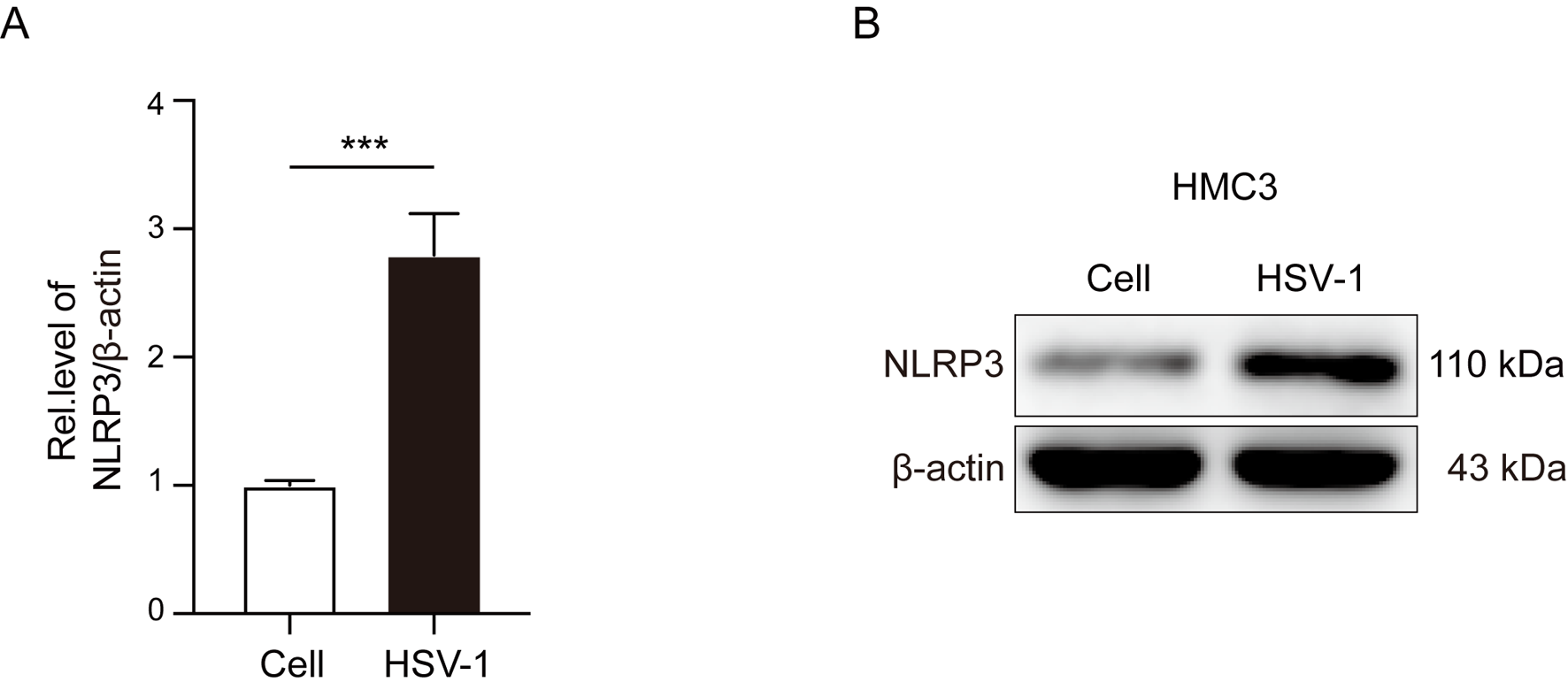

Supplement: Supplementary Figure 2 — HSV-1 induces NLRP3 expression. (A) Relative qRT-PCR analysis of NLRP3 mRNA levels in BV2 cells infected with HSV-1(MOI = 5, 24 hpi). (B) HMC3 cells were infected with HSV-1 (MOI = 5) for 24 h, NLRP3 was detected by Western blot. All data are presented as mean ± SD, Student’s t-test, ***P < 0.001. [file Image_2.TIF]

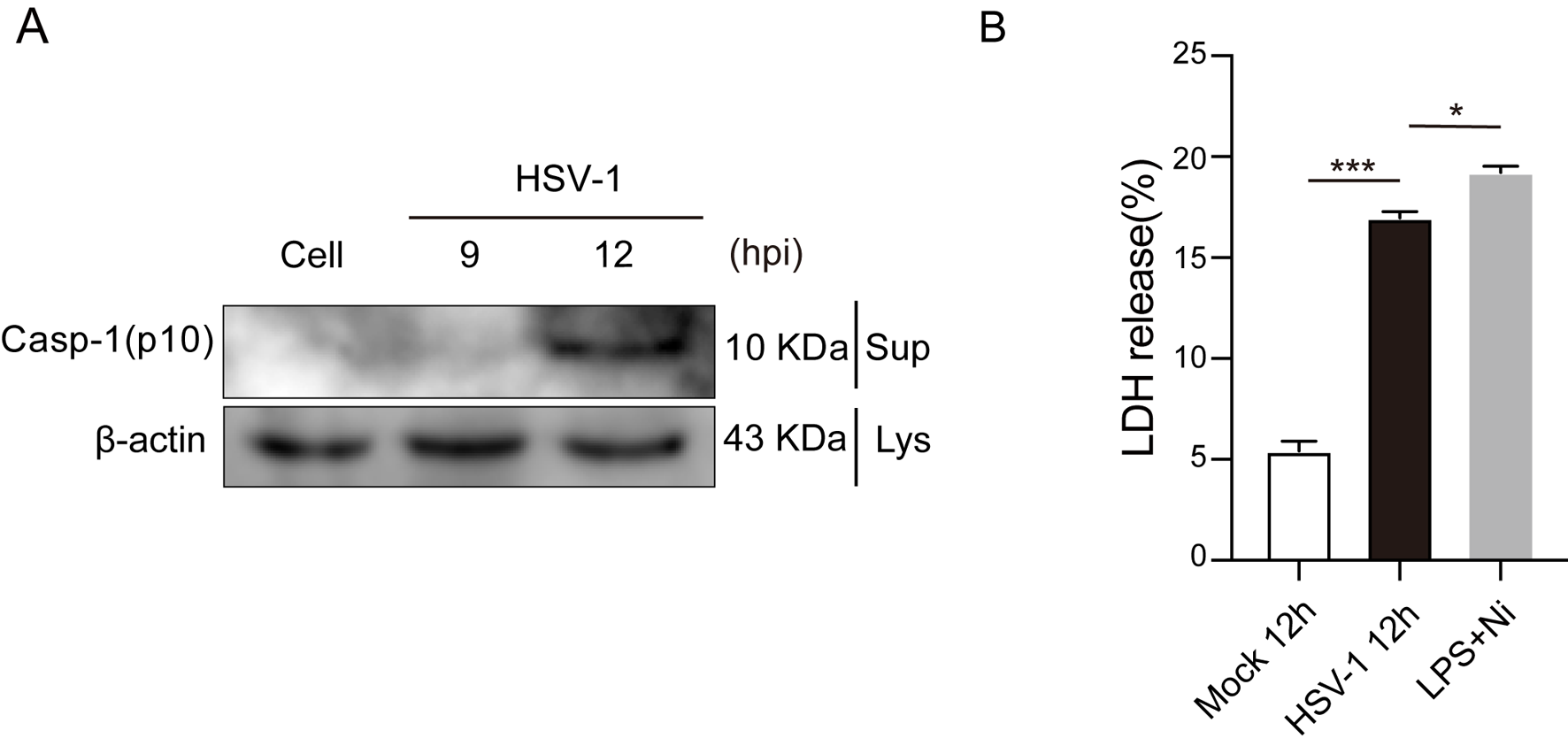

Supplement: Supplementary Figure 3 — HSV-1 began to induce microglia pyroptosis at 12 hpi. (A) Immunoblot analysis of supernatants (Sup) of BV2 infected by HSV-1 (MOI = 5) for the indicated time points by the caspase-1 (p10). (B) LDH release was measured in the supernatant from simulated Mock and HSV-1 infected BV2 (12 hpi). All data are presented as mean ± SD, Student’s t-test, *P < 0.05, ***P < 0.001. [file Image_3.TIF]

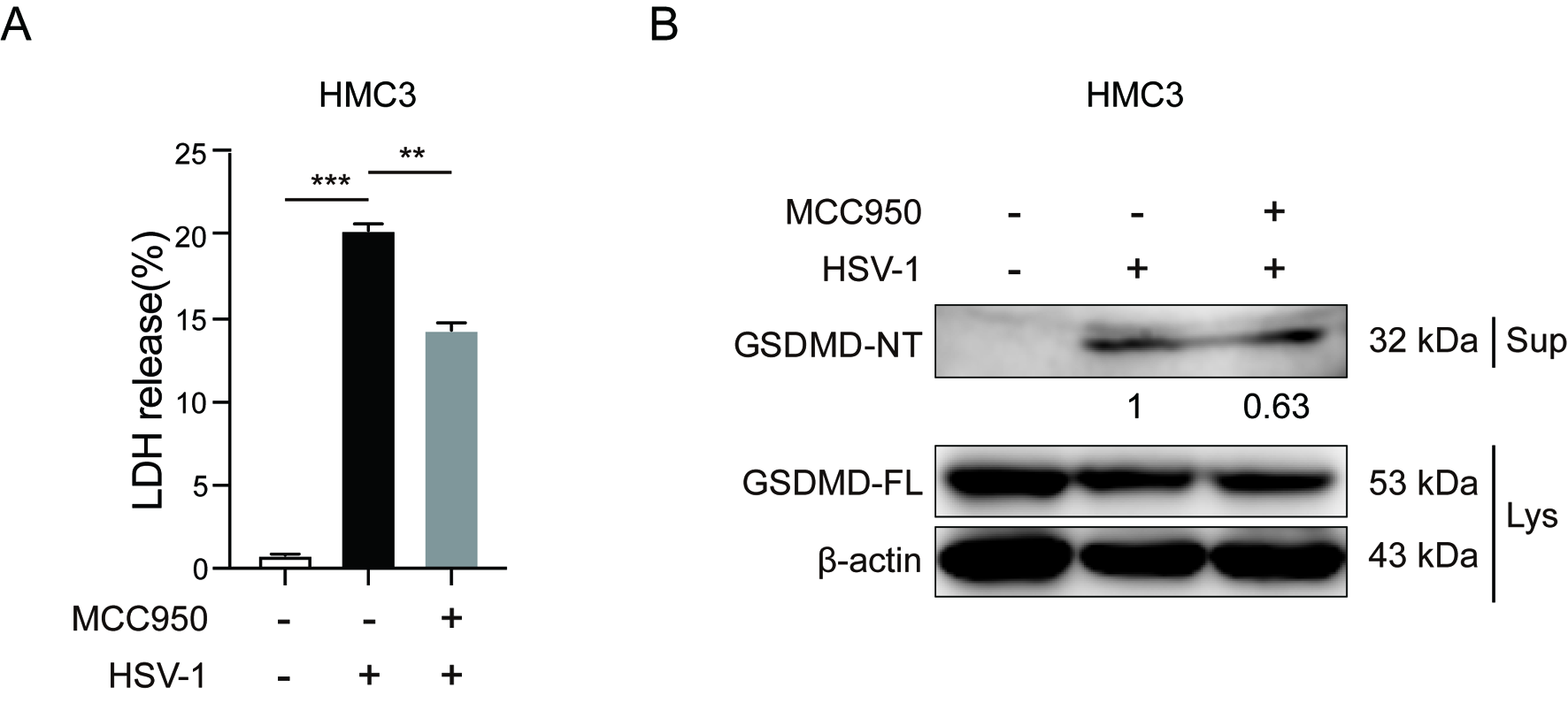

Supplement: Supplementary Figure 4 — MCC950 inhibits HSV-1 induced pyroptosis in HMC3 cells. (A) HMC3 cells pretreated with MCC950 (5 μg/mL) for 1 h and LDH release was measured in supernatant derived from MCC950, HSV-1 (MOI = 5) and HSV-1 + MCC950 (5 μg/mL). (B) HMC3 cells were treated as (A) in the presence of MCC950, and the total DNA and protein of HSV-1 were extracted for analysis. Immunoblot analysis of extracts of BV2s by the indicated antibodies. All data are presented as mean ± SD, Student’s t-test, **P < 0.01, ***P < 0.001. [file Image_4.TIF]
