# Supplementary material for: Herpes Simplex Virus 1 Induces Microglia Gasdermin D-Dependent Pyroptosis Through Activating the NLR Family Pyrin Domain Containing 3 Inflammasome
Source: Front Microbiol. 2022 Mar 21;13:838808. doi: 10.3389/fmicb.2022.838808 (PMC8978634; doi:10.3389/fmicb.2022.838808)
Supplement: Supplementary file 5 [file Table_1.DOCX]

**Supplernentary table 1**

The primer sequences for quantitative real-time PCR (qRT-PCR).

| Gene | Forward (5′-3′ sequence) | Reverse (5′-3′ sequence) |
| --- | --- | --- |
| IL-1β | AATGCCACCTTTTGACAGTGATG | AGCTTCTCCACAGCCACAAT |
| ICP0 | CCCACTATCAGGTACACCAGCTT | CTGCGCTGCGACACCTT |
| NLRP3 | ATTACCCGCCCGAGAAAGG | TCGCAGCAAAGATCCACACAG |
| IL-18 | GTGAACCCCAGACCAGACTG | CCTGGAACACGTTTCTGAAAGA |
| β-actin | GTGACGTTGACATCCGTAAAGA | GCCGGACTCATCGTACTCC |
